# Single isocenter SRS using CAVMAT offers improved robustness to commissioning and treatment delivery uncertainty compared to VMAT

**DOI:** 10.1002/acm2.13248

**Published:** 2021-06-24

**Authors:** Edward T. Cullom, Yuqing Xia, Kai‐Cheng Chuang, Zachary W. Gude, Yana Zlateva, Justus D. Adamson, William M. Giles

**Affiliations:** ^1^ Medical Physics Graduate Program Duke University Durham North Carolina USA; ^2^ Department of Radiation Oncology Duke University Medical Center Durham North Carolina USA; ^3^ Medical Physics Graduate Program Duke Kunshan University Suzhou China

**Keywords:** CAVMAT, single isocenter, SRS, VMAT

## Abstract

**Purpose:**

In this study, we evaluate and compare single isocenter multiple target VMAT (SIMT) and Conformal Arc Informed VMAT (CAVMAT) radiosurgery's sensitivity to uncertainties in dosimetric leaf gap (DLG) and treatment delivery. CAVMAT is a novel planning technique that uses multiple target conformal arcs as the starting point for limited inverse VMAT optimization.

**Methods:**

All VMAT and CAVMAT plans were recalculated with DLG values of 0.4, 0.8, and 1.2 mm. DLG effect on V_6Gy_[cc], V_12Gy_[cc], and V_16Gy_[cc], and target dose was evaluated. Plans were delivered to a Delta^4^ (ScandiDos, Madison, WI) phantom and gamma analysis performed with varying criteria. Log file analysis was performed to evaluate MLC positional error. Sixteen targets were delivered to a SRS MapCHECK (Sun Nuclear Corp., Melbourne, FL) to evaluate VMAT and CAVMAT's dose difference (DD) as a function of DLG.

**Results:**

VMAT's average maximum and minimum target dose sensitivity to DLG was 9.08 ±3.50%/mm and 9.50 ± 3.30%/mm, compared to 3.20 ± 1.60%/mm and 4.72 ± 1.60%/mm for CAVMAT. For VMAT, V_6Gy_[cc], V_12Gy_[cc], and V_16Gy_[cc] sensitivity was 35.83 ± 9.50%/mm, 34.12 ± 6.60%/mm, and 39.23 ± 8.40%/mm. In comparison, CAVMAT's sensitivity was 23.19 ± 4.50%/mm, 22.45 ± 4.40%/mm, and 24.88 ± 4.90%/mm, respectively. Upon delivery to the Delta^4^, CAVMAT offered superior dose agreement compared to VMAT. For a 1%/1 mm gamma analysis, VMAT and CAVMAT had a passing rate of 94.53 ± 4.40% and 99.28 ± 1.70%, respectively. CAVMAT was more robust to DLG variation, with the SRS MapCHECK plans yielding an absolute average DD sensitivity of 2.99 ± 1.30%/mm compared to 5.07 ± 1.10%/mm for VMAT. Log files demonstrated minimal differences in MLC positional error for both techniques.

**Conclusions:**

CAVMAT remains robust to delivery uncertainties while offering a target dose sensitivity to DLG less than half that of VMAT, and 65% of that of VMAT for V_6Gy_[cc], V_12Gy_[cc], and V_16Gy_[cc]. The superior dose agreement and reduced sensitivity of CAVMAT to DLG uncertainties indicate promise as a robust alternative to VMAT for SIMT SRS.

## 1 INTRODUCTION

Brain metastases are a common diagnosis for many cancer patients and can be treated with various stereotactic radiosurgery (SRS) techniques.^1^ Linear accelerator‐based SRS is traditionally carried out with each metastasis being treated individually using cones, noncoplanar conformal fields, or dynamic conformal arcs with MLCs.^2^, ^3^, ^4^ In addition to traditional techniques, single isocenter VMAT for simultaneous SRS to multiple targets has also grown in prevalence. This technique has the advantage of increasing the treatment delivery efficiency, and numerous reports have been made regarding planning techniques,^5^, ^6^, ^7^, ^8^, ^9^, ^10^, ^11^ immobilization and quality assurance,^12^, ^13^, ^14^, ^15^, ^16^, ^17^ as well as clinical outcomes.^18^, ^19^, ^20^

In addition to the stringent immobilization requirements for single isocenter multiple target (SIMT) radiosurgery, another challenge is accurate dosimetric modeling and treatment delivery. The dosimetry and modeling of small MLC openings, commonly used in multitarget radiosurgery, is particularly challenging as charged particle equilibrium may fail and targets may be treated off‐axis. Disequilibrium paired with steep dose gradients complicates dosimetric measurements, further increasing existing uncertainty. Additionally, the dosimetric leaf gap (DLG) accounts for beam transmission through the rounded leaf edges of the MLCs, and the DLG configuration may introduce uncertainty as in some planning systems a single value must be chosen to suit all types of targets, treatment geometries, and MLC geometries. Optimal DLG values can be dependent on the type of treatment plan and beam model utilized.^21^ Some institutions have even reported difficulty in identifying a single DLG that is representative for all radiosurgery targets, necessitating the need to split targets into multiple isocenter groups, partially negating the time saving benefit of single isocenter treatments.^9^, ^22^

Additional uncertainty may arise from the treatment delivery. The physical motion of a given leaf pair may deviate from optimal MLC motion in the treatment planning system and similar deviations in gantry angle may occur during arc delivery. Prior studies have demonstrated that multitarget radiosurgery techniques are uniquely susceptible to dosimetric effects from treatment delivery uncertainties, especially when targets are small and/or distant from the isocenter.^15^, ^16^, ^17^ Furthermore, there are few commercial QA tools with sufficient spatial resolution and comprehensive dosimetry to quantify the dosimetric effect of treatment delivery discrepancies. While prior studies have investigated the clinical dosimetric effect of mechanical discrepancies in gantry, couch, and collimator angles,^15^ as well as patient rotational setup uncertainties,^16^, ^17^ in this study, we expand upon prior analyses to include uncertainties in MLC positions and gantry angle as measured during treatment delivery.

In summary, this study aims to evaluate and compare the dosimetric sensitivity of SIMT VMAT and CAVMAT, a novel treatment planning technique, to uncertainties in DLG value in the planning system and mechanical treatment delivery errors.

Conformal Arc Informed VMAT (CAVMAT) is a specialized SIMT VMAT technique that has recently been proposed to overcome one of the main challenges of SIMT VMAT for SRS. Due to the number of targets, geometry, and modulation, the MLCs in SIMT VMAT plans are often unable to block between targets sharing the same leaf pair.^23^, ^24^ Some proposed methods to address this challenge include an algorithm to identify the optimal collimator angle per arc,^25^ as well as an algorithm to identify the full arc geometry with the least unblocked area per arc.^26^ However, even with these strategies, this limitation cannot always be avoided, especially as the number of targets increases; and often the best alternative is the use of structures and constraints in the inverse optimization. In contrast, CAVMAT is a hybrid method between dynamic conformal arcs and VMAT that aims to provide optimal collimation by ensuring targets do not share the same leaf pair, and which has been described in detail previously.^24^ We hypothesize that the CAVMAT treatment planning technique will be less sensitive than VMAT to configuration and delivery uncertainties due to the simplified and MLC motion.

## 2 METHODS

### 2.1 Overview

This comparison of CAVMAT and SIMT radiosurgery VMAT is divided into three parts: (a) evaluation of robustness to uncertainties in DLG, (b) evaluation of robustness to mechanical uncertainties at treatment delivery, and (c) a series of measurements with pretreatment QA devices to evaluate the combination of dose calculation and treatment delivery uncertainties. For the evaluation of robustness to uncertainties in DLG, we varied the beam model DLG, recalculated treatment plans with fixed monitor units, and quantified the dosimetric impact. For the evaluation of robustness to mechanical uncertainties at treatment delivery, we recalculated the delivered treatment plans using the machine parameters recorded in the trajectory log files. For the combined analysis, we compared calculated and measured dose with two pretreatment QA devices: Delta4 (ScandiDos, Madison WI), and SRS MapCHECK (SunNuclear, Melbourne FL); the SRS MapCHECK comparison focuses on individual targets and includes dose calculations at each DLG setting.

### 2.2 Treatment plans

Ten previously treated SIMT VMAT cases were selected with each case including three to seven brain metastases. Only cases that were prescribed a single fraction were utilized, restricting target size to ≤2 cm in equivalent sphere diameter. In total, 45 targets were included and analyzed in this study. Varian Eclipse 15.6 (Varian Medical Systems) was used for the treatment planning and all plans were calculated using Anisotropic Algorithm (AAA) with a 1‐mm dose voxel size. A TrueBeam STx linear accelerator with HDMLC and 6XFFF photons was used for all treatment plans, treatment deliveries, and QA measurements. Each case was prepared following planning techniques described previously^10^, ^11^, ^16^ by an ABR certified physicist with experience in clinical VMAT SRS treatment planning and was approved by the attending physician. All targets were prescribed 20 Gy to simplify the reported statistics and to make the results more uniform and significant. Of the 10 VMAT cases included in this study, three were re‐planned to ensure that all targets received the prescription dose of 20 Gy (also performed by an ABR certified physicist with SRS planning experience). For each patient geometry, target volume, equivalent sphere radius, and distance from radiation isocenter were measured.

All VMAT plans were re‐planned with CAVMAT, with the same planning system and dose calculation algorithm. CAVMAT is comprised of the following three main steps: target subgrouping, field weight optimization, and limited inverse optimization. CAVMAT involves dividing targets into subgroups at each arc geometry, which are treated using their own dynamic conformal arc or subarc. Target subgroups are selected such that a collimator angle can be found to appropriately block between all targets within the subgroup for the entire arc. Further details regarding the CAVMAT technique are provided in the supplemental materials and in a prior study.^24^

### 2.3 Robustness to DLG uncertainties

All plans were calculated using varying DLG values of 0.4, 0.8, and 1.2 mm (with no other differences in the beam model). Cases were first calculated with a 0.4‐mm DLG, establishing a baseline for subsequent comparison. The same MU per arc was used when calculating all DLG plans and plans were not re‐normalized. For all plans, the following metrics were evaluated: volume of healthy tissue receiving 6 Gy (V_6_ _Gy_[cc]), V_12_ _Gy_[cc], V_16_ _Gy_[cc], minimum dose, mean dose, maximum dose, conformity index, and limiting coverage (D_99%_[%]) for all 45 targets. Conformity index is defined as the volume enclosed in the prescription isodose surface (*V_1_*) divided by the total target volume (*V_t_*).^27^

(1) $$\begin{array}{c} CI=\frac{V_{I}}{V_{t}} \end{array}.$$

The percent change in dose statistics from 0.4 to 0.8 mm and 0.4 to 1.2 mm was calculated and normalized by the difference in DLG value, using 0.4 mm as a baseline. The normalized percent change for different DLG values and dose statistics was averaged and reported as DLG sensitivity in units %/mm.

### 2.4 Robustness to mechanical delivery uncertainties

The VMAT and CAVMAT plans were delivered, and the MLC positions were automatically stored as log files in the TrueBeam system (Varian Medical Systems, Palo Alto, CA).^28^ An in‐house script written in Python v.3.7 (Python Software Foundation, Wilmington, DE) utilizing Pylinac was used to extract delivered MLC and gantry positions for each arc control point from the log files.^29^ A copy of the original plan was created where the original MLC, and gantry positions were overwritten with the positions recorded in the log files. Dose was recalculated for the updated plans to construct an accurate representation of the delivered plan. The delivered plans were calculated with the same total MU, AAA algorithm, and dose grid size as the original treatment plans. The original VMAT and CAVMAT plans were compared to the delivered plans to assess the dosimetric change and general sensitivity to treatment delivery. Change in relevant clinical dose statistics (such as those noted in the DLG section above) was evaluated. Relative MLC positional error at each control point was also quantified from the trajectory log files as the absolute difference between planned and delivered MLC position.

### 2.5 Combined uncertainty analysis via pretreatment QA

In this study, two different detectors were used to validate dose discrepancies between the planned and delivered VMAT and CAVMAT plans. The Delta^4^ and SRS MapCHECK phantoms were chosen for this study and complement each other well, as the Delta^4^ is able to evaluate 3D volumetric doses while the SRS MapCHECK can evaluate 2D dose at a superior resolution. Couch rotations were not used for either the Delta^4^ or SRS MapCHECK verification plans to ensure that all targets were sufficiently measured by the detectors.

The 10 VMAT and CAVMAT plans were recalculated on the Delta^4^ phantom geometry, using a DLG of 0.4 mm and were subsequently delivered. Calculated and measured dose from the Delta^4^ treatment delivery was compared using Gamma Index with increasingly strict passing criteria (3%/1 mm, 2%/1 mm, and 1%/1 mm). The percent of measured points with absolute difference between planned and delivered dose being greater than 3%, 2%, and 1% was also calculated.

In total, the 10 plans evaluated in this study consisted of 45 targets. Sixteen of the 45 total targets were selected, with at least one target selected from each plan. These sixteen targets were delivered to a SRS MapCHECK QA device within the StereoPHAN phantom (Sun Nuclear, Melbourne, FL). For each target, a QA verification plan was created with the target centered on the diode array. Dose was calculated to the phantom and QA device for all DLG settings and compared to the measured dose distribution. The dose difference was determined for all measurement points with dose above a threshold of 50% of the maximum measured dose. This threshold was chosen to avoid accounting for low dose regions with very high passing rates, which may obscure finer details and discrepancies.

## 3 RESULTS

### 3.1 Robustness to DLG uncertainties

Across all 10 plans, CAVMAT was found to more robust than VMAT to DLG variation and its corresponding impact on normal tissue and target doses. CAVMAT's average DLG sensitivity to changes in V_6_ _Gy_[cc], V_12_ _Gy_[cc], and V_16_ _Gy_[cc] was 23.19 ± 4.50%/mm, 22.45 ± 4.40%/mm, and 24.88 ± 4.90%/mm, respectively, compared to 35.83 ± 9.50%/mm, 34.12 ± 6.60%/mm, and 39.22 ± 8.40%/mm for VMAT. Similarly, CAVMAT presented an average DLG sensitivity to changes in target maximum, mean, and minimum dose of 3.20 ± 1.60%/mm, 3.5 ± 0.40%/mm, and 4.72 ± 1.60%/mm, respectively. In comparison, VMAT presented an increased DLG sensitivity to the same target doses of 9.08 ± 3.50%/mm, 9.22 ± 3.20%/mm, and 9.50 ± 3.30%/mm.

**Fig. 1:**
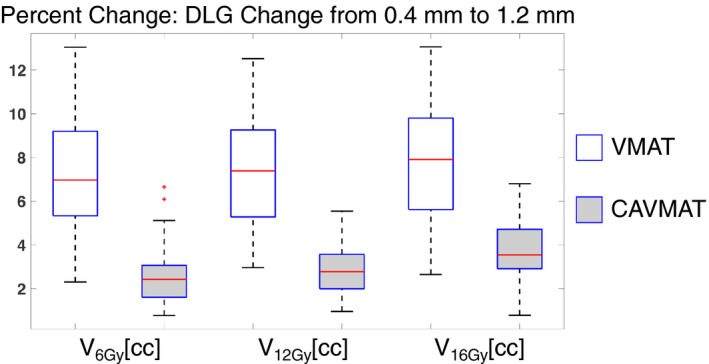
Reduced sensitivity of CAVMAT to changes in V_6 Gy_[cc], V_12_ _Gy_[cc], and V_16_ _Gy_[cc], for varying DLG.

**Fig. 2:**
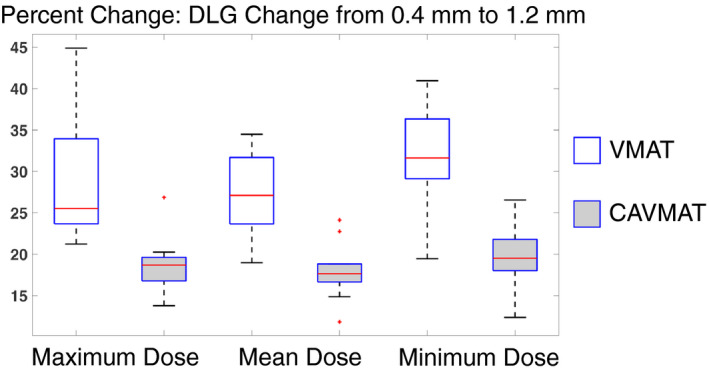
VMAT increases the maximum dose to all targets significantly more than CAVMAT (*p* = 2.17 × 10^−12^).

The average limiting coverage (D_99%_[%]) for all targets was evaluated at all DLG values. For an increasing DLG, coverage is expected to increase, indicated by shifts in the DVH. For the same change in DLG values, CAVMAT's target coverage sensitivity to DLG was nearly half that of VMAT (4.90%/mm compared to 8.93%/mm). To contextualize DLG sensitivity, the raw change in dosimetric indices when the DLG is changed from 0.4 to 1.2 mm is displayed in Figs. 1 and 2 for both VMAT and CAVMAT. For a DLG change of 0.4 to 1.2 mm, VMAT's healthy tissue V_6_ _Gy_[cc], V_12_ _Gy_[cc], and V_16_ _Gy_[cc] increased on average by 7.27 ± 2.80%, 7.38 ± 2.60%, and 7.60 ± 2.60%, respectively. In comparison, CAVMAT's sensitivity was 2.56 ± 1.30%, 2.80 ± 1.10%, and 3.78 ± 1.30%, more than half the sensitivity of VMAT. Similarly, the target maximum, mean, and minimum dose of the VMAT plans increased on average by 28.67 ± 7.60%, 27.30 ± 5.30%, and 31.38 ± 6.70%, respectively, compared to 18.55 ± 3.60%, 17.96 ± 3.50%, and 19.91 ± 3.90% for CAVMAT. Both VMAT and CAVMAT DLG sensitivities were not dependent on target distance from isocenter, radius, or volume.

### 3.2 Robustness to mechanical delivery uncertainties

Similar to DLG uncertainty, CAVMAT was more robust than VMAT to dosimetric changes following treatment delivery. Following delivery and log file analysis, the average V_6_ _Gy_[cc], V_12_ _Gy_[cc], and V_16_ _Gy_[cc] of the 10 VMAT plans increased by 0.93 ± 1.40%, 0.90 ± 1.40%, and 1.23 ± 1.50%, respectively. In comparison, the V_6 Gy_[cc] of the CAVMAT plans actually decreased by 0.03 ± 0.10%. V_12 Gy_[cc] and V_16_ _Gy_[cc] increased by 0.14 ± 0.20% and 0.28 ± 0.20%, respectively. Following delivery, VMAT exhibit a slightly increased average maximum, mean, and minimum target dose of 0.53 ± 0.50%, 0.52 ± 0.50%, and 0.53 ± 0.60%, respectively. CAVMAT also exhibited a small increase in average maximum, mean, and minimum dose of 0.16 ± 0.20%, 0.11 ± 0.10%, and 0.07 ± 0.10%. The CAVMAT plans were also found to be more conformal than their VMAT counterparts. CAVMAT's average conformity index increased by 0.79 ± 0.70% (1.38 ± 0.20 to 1.39 ± 0.20) compared to an increase of 3.74 ± 3.40% (1.48 ± 0.20 to 1.54 ± 0.20) for VMAT. The CAVMAT plans offered superior target coverage compared to VMAT and also better maintained this coverage following delivery. VMAT's average target coverage increased by 0.15 ± 0.20% (99.50%–99.65%), compared to a change of 0.01 ± 0.30% (99.65%–99.67% coverage) for CAVMAT. Both VMAT and CAVMAT were robust to MLC and gantry positional error. VMAT's absolute average MLC positional error was 0.002 ± 0.01 mm, while the absolute average gantry positional error was 0.02 ± 0.30°. The absolute average MLC and positional error for CAVMAT was 0.002 ± 0.02 mm and 0.02 ± 0.30°, respectively.

### 3.3 Combined uncertainty analysis via pretreatment QA

**Fig. 3:**
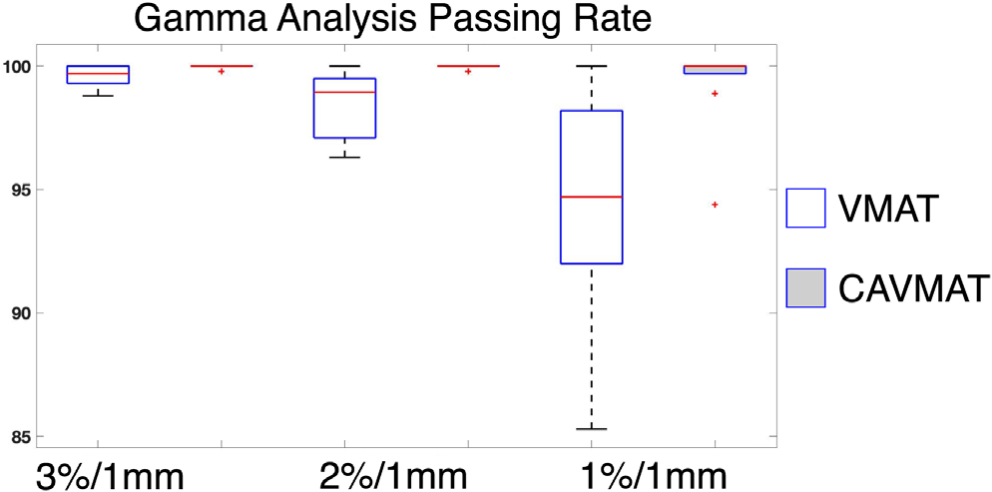
Percent of Delta^4^ detector pixels with a gamma index <1 with varying analysis criteria for the 10 radiosurgery plans.

Gamma analysis was performed for the Delta^4^ measurements. For a 3%/1 mm, 2%/1 mm, and 1%/1 mm criteria, VMAT yielded an average passing rate of 99.61 ± 0.40%, 98.54 ± 1.30%, and 94.53 ± 4.40%, respectively. For the same criteria, CAVMAT's passing rate was 99.98 ± 0.10%, 99.98 ± 0.10%, and 99.28 ± 1.70%. As displayed below in Fig. 3, the difference between VMAT and CAVMAT was increasingly apparent as the gamma index comparison criteria were tightened.

**Fig. 4:**
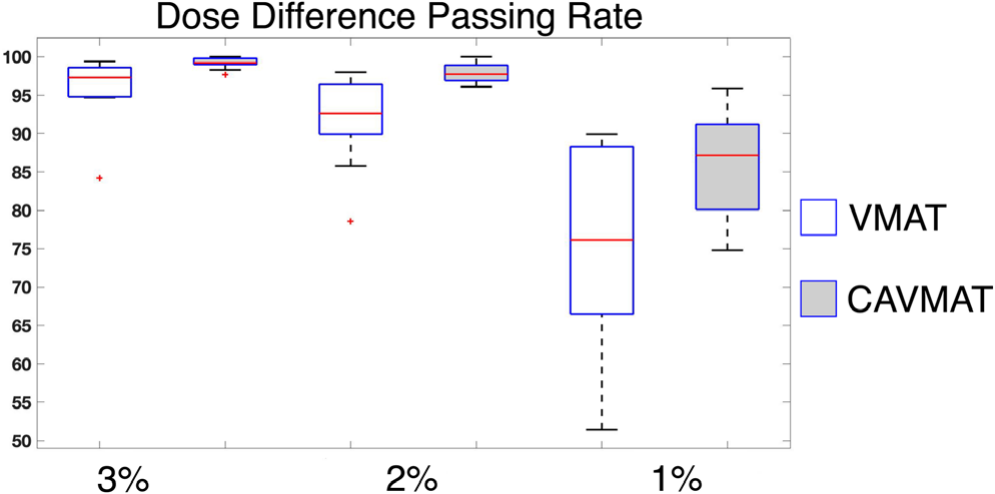
Percent of Delta^4^ detector pixels with dose difference was less than 3%, 2%, and 1%, for the 10 radiosurgery plans.

The raw difference between calculated and measured dose with the Delta^4^ was also quantified for both VMAT and CAVMAT and is displayed in Fig. 4. For dose difference criteria of 3%, 2%, and 1%, the average passing rate of the VMAT plans was 96.03 ± 4.40%, 91.74 ± 5.90%, and 74.78 ± 13.0%, respectively. In comparison, CAVMAT offered a greater dose agreement, with passing rates of 99.17 ± 0.70%, 97.91 ± 1.40%, and 86.16 ± 6.40%.

**Fig. 5:**
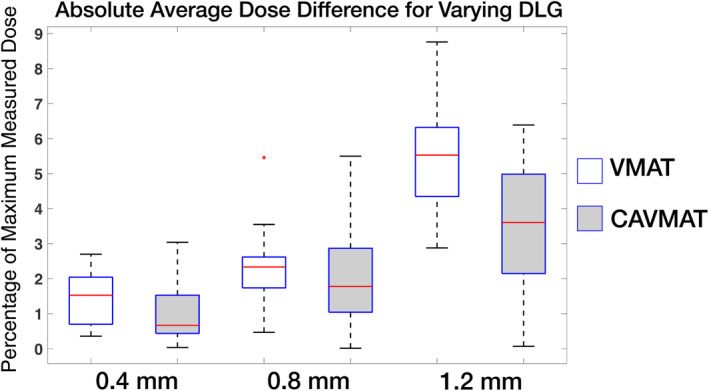
SRS MapCHECK dose difference from varying DLG for all measured points above a 50% threshold.

The absolute average dose difference for the 16 VMAT and CAVMAT targets delivered to the SRS MapCHECK was evaluated for differing DLG values. All three calculated isodose distributions (0.4‐, 0.8‐, and 1.2‐mm DLG) were compared against a delivered 0.4‐mm DLG baseline. Dose difference values are a percentage of the maximum measured dose. VMAT yielded an absolute average dose difference sensitivity of 5.07 ± 1.10%/mm, compared to a sensitivity of only 2.99 ± 1.30%/mm for CAVMAT. For instance, for a calculated DLG of 0.4, 0.8, and 1.2 mm, with a 50% threshold, VMAT presented an absolute average dose difference of 1.46 ± 0.70%, 2.32 ± 1.20%, and 5.52 ± 1.60%, respectively. For the same DLG values, CAVMAT yielded a reduced absolute average dose difference of 1.05 ± 0.90% (*P* = 0.003, compared to VMAT), 1.94 ± 1.40% (*P* = 0.06) and 3.44 ± 1.90% (*P* = 7.7 × 10^−4^), respectively. The effect of DLG on absolute average dose difference for both techniques is displayed in Fig. 5.

## 4 DISCUSSION

V_6 Gy_[cc], V_12_ _Gy_[cc], and V_16_ _Gy_[cc] have been associated with radionecrosis and neurocognitive decline and are often used to assess the relative risk for SRS.^30^, ^31^, ^32^ Thorough understanding of dose‐volume parameters in the treatment planning system is crucial for the accurate prediction of posttreatment complications. Lower dose values, such as 6 and 12 Gy, lie in the penumbra region of the beam, where there is a greater dose uncertainty.^33^ The clinical importance of these dose‐volume parameters highlights the necessity for treatment planning techniques that are less sensitive to these uncertainties.

Numerous studies have investigated the commissioning process of determining the DLG value using set field sizes and the sweeping gap technique. In most cases, the DLG value calculated at commissioning does not provide a sufficiently close match between measured and calculated dose, necessitating a further correction of the DLG.^21^, ^34^, ^35^, ^36^ In some instances, failing to correct the calculated DLG value may lead to a difference in measured and calculated dose on the order of 5%.^34^ While differing DLG values may be required for different types of radiation treatment, some institutional studies have even reported difficulty in determining a single DLG that is sufficient for all of the institutions' radiosurgery cases.^9^, ^22^

While different MLC systems and photon energies are expected to result in differing optimized DLG values, large differences are present even for the same energy and MLC system. For a HDMLC system and 6MV‐FFF photon beam, Yao et al. found an optimized DLG value of 0.3 mm by using test fields while Kim et al. optimized the DLG to 0.9 mm via the sweeping gap technique.^35^, ^37^ Kim et al. also demonstrated that the DLG may be optimized using film or ion chamber measurements for site specific treatment plans, such as spine SBRT or intracranial SRS.^37^ Some studies have reported even larger DLG values, with Kielar et al., determining a DLG of 1.7 mm for VMAT.^34^ In the context of our results, this range in DLG values (0.3–1.7 mm) when applied to single isocenter radiosurgery could correspond to a dose difference (between the maximum and minimum DLG values) for the mean target dose of approximately 12.9% with VMAT and 7.1% for CAVMAT. This highlights the necessity of a careful beam commissioning process for this technique.

The potential for substantial dose discrepancies paired with the difficulty in determining a single DLG for all target geometries necessitates a treatment technique that is resistant to DLG variation. If systematic error exists in the choice of DLG, or for cases where the optimal DLG varies between plans or targets within a single plan, the dosimetric impact may be reduced by using the CAVMAT treatment planning technique. Our results indicate that CAVMAT is less sensitive to DLG errors compared to a conventional VMAT technique. CAVMAT's V_6 Gy_[cc], V_12_ _Gy_[cc], and V_16_ _Gy_[cc] sensitivity to changes in DLG was only 63%–66% of VMAT's sensitivity. Similarly, CAVMAT's sensitivity of maximum, mean, and minimum target dose to DLG was only 44%–62% of the VMAT's sensitivity. Thus, for these cases when the value of the DLG was modified, CAVMAT was more effective in maintaining the planned doses to healthy tissues, coverage, and dose applied to each target. In this study, DLG sensitivity was found to be independent of target radius, volume, and distance from isocenter.

While errors may arise from improper beam configuration, uncertainty may also be inherently present in treatment delivery. In some scenarios, the physical MLC motion may differ from the intended motion defined in the treatment plan. Variation in MLC motion can be due to gravitational effects and leaf pair extension and contraction speed. Previous studies have used a similar log file analysis methodology to investigate the dosimetric impact of MLC position and gantry rotation errors; however, the primary focus was on head and neck or prostate cancer.^38^, ^39^ While our study analyzes MLC positional error in a similar manner, the primary focus is multiple brain metastases, treated with a single isocenter. In our study, MLC positional error for both the VMAT and CAVMAT plans was found to be small and comparable, demonstrating that the linear accelerator is capable of delivering CAVMAT plans as effectively as VMAT plans.

In our study, measurements with the SRS MapCHECK were used to compare measured and calculated dose with different DLG values, and we reported the mean dose difference for all measurement points above a 50% threshold. Conceptually, this is somewhat comparable to the dosimetric effect of DLG on mean target dose. The DLG sensitivity of the mean target dose for VMAT was found to be 9.22 ± 3.20%/mm, compared to the mean dose difference from SRS MapCHECK of 5.07 ± 1.10%/mm; thus, the actual clinical effect was found to be greater than the effect measured by the pretreatment QA device.

While the differences in delivered target doses and dose‐volume statistics were not large, gamma analysis demonstrated that the CAVMAT technique provided a superior agreement between planned and delivered dose than VMAT. The CAVMAT technique offered a superior gamma analysis passing rate for each criteria, particularly the 1%/1‐mm test. The dose difference passing rate followed a similar trend, demonstrating that CAVMAT resulted in better dose agreement than VMAT. The superior dose agreement of CAVMAT was consistent for a varying DLG, as exemplified by the SRS MapCHECK. The SRS MapCHECK analysis indicates that even in suboptimal DLG scenarios, CAVMAT can better maintain dose agreement and may better maintain plan quality than conventional techniques. The difference in agreement for the measurement may be related to the sensitivity to beam configuration for the two types of plans, given the minimal effect observed from the log file analysis.

There are other beam configuration parameters and general treatment errors that were not evaluated in this study. Future studies may investigate planning and delivery sensitivity to changing focal spots and the determination of MLC transmission factors. Future work may involve evaluating the sensitivity to beam configuration errors and delivery errors of other common or emerging radiosurgery techniques, such as Elements (Brainlab, Munich Germany) and HyperArc (Varian Medical Systems, Palo Alto CA). Brainlab Elements is an automated planning system which utilizes a single isocenter to treat multiple targets.^40^ Elements utilizes less complex MLC trajectories, similar to CAVMAT, and may also feature a reduced sensitivity to configuration and delivery errors. While Elements is conceptually similar to CAVMAT, the inherent differences in treatment planning systems and beam modeling warrant further evaluation to determine whether the results found for CAVMAT also apply to Elements. HyperArc also utilizes a single isocenter and employs an automated process to determine couch and collimator angles, as well as beam arrangement^8^, ^18^; the automated process and optimization of treatment geometry may also reduce sensitivity to DLG variation.

## 5 CONCLUSION

Employing techniques with a reduced sensitivity to DLG variation is crucial as it is unlikely that the DLG selected will be optimal for all treatment sites, especially in SIMT applications where multiple targets are treated off central axis. For instance, for SIMT SRS using VMAT, V_16 Gy_[cc] was found to be the most sensitive to uncertainty in DLG at 39.23 ± 8.40%/mm. Substantial sensitivity was also observed for V_6_ _Gy_[cc] and V_12_ _Gy_[cc]. Sensitivity of target dose was about four times less, with a minimum dose sensitivity of 9.50 ± 3.30%/mm. Delivery and analysis via SRS MapCHECK indicated a similar sensitivity in the high dose region.

Compared to VMAT, CAVMAT was only half to two thirds as sensitive to uncertainties in choice of DLG. The robustness of CAVMAT was reinforced by QA measurements, where gamma analysis further confirmed the reduced sensitivity to DLG configuration and treatment delivery errors as well as the superior dose agreement of CAVMAT. The reduced MLC and gantry uncertainty of CAVMAT demonstrates that CAVMAT plans are just as feasible as VMAT to deliver but also offer greater dose agreement and reduced sensitivity to DLG. Thus, the CAVMAT techniques offer a promising approach to minimize the impact of DLG configuration and delivery uncertainties, potentially improving overall treatment quality.

## CONFLICT OF INTEREST

No conflicts of interest to report.

## Supporting information


**Data S1**. Details of CAVMAT treatment planning technique.

## Data Availability

The data that support the findings of this study are available on request from the corresponding author. The data are not publicly available due to privacy or ethical restrictions.
