# Supplementary material for: Single isocenter SRS using CAVMAT offers improved robustness to commissioning and treatment delivery uncertainty compared to VMAT
Source: J Appl Clin Med Phys. 2021 Jun 24;22(7):36–43. doi: 10.1002/acm2.13248 (PMC8292691; doi:10.1002/acm2.13248)
Supplement: Supplementary file 1 — Data S1. Details of CAVMAT treatment planning technique. [file ACM2-22-36-s001.pdf]

Supplemental Material

In this study 45 targets across 10 different plans were evaluated to compare CAVMAT and VMAT. A summary of the targets' volume, radius, and distance from isocenter is included below in table 1. All targets featured in table 1 were given 1 mm margins.

Table 1: Summary of the 45 targets used in this study

Target Overview							
**Target #**	Volume (cc)	Radius (cm)	Distance from isocenter (cm)	Target #	Volume (cc)	Radius (cm)	Distance from isocenter (cm)
**1**	1.02	0.60	4.40	**24**	0.14	0.30	5.53
**2**	0.13	0.30	6.07	**25**	0.37	0.45	5.09
**3**	0.30	0.40	6.37	**26**	0.34	0.45	3.28
**4**	0.21	0.35	3.21	**27**	0.73	0.55	5.54
**5**	0.14	0.30	2.10	**28**	0.44	0.45	2.69
**6**	0.17	0.35	2.30	**29**	0.34	0.45	3.28
**7**	0.15	0.35	0.65	**30**	0.23	0.40	5.21
**8**	0.42	0.45	4.10	**31**	0.07	0.50	5.46
**9**	0.27	0.40	1.96	**32**	0.17	0.70	3.37
**10**	0.28	0.40	4.49	**33**	0.04	0.40	6.41
**11**	0.32	0.45	8.25	**34**	0.39	0.45	1.74
**12**	0.13	0.30	5.32	**35**	1.13	0.65	5.50
**13**	0.28	0.40	7.52	**36**	2.64	0.85	4.10
**14**	0.51	0.50	6.31	**37**	0.19	0.35	4.50
**15**	0.60	0.50	9.97	**38**	0.59	0.50	1.61
**16**	0.41	0.45	3.13	**39**	0.09	0.30	3.63
**17**	1.15	0.65	7.45	**40**	0.25	0.40	3.86
**18**	0.24	0.40	7.68	**41**	0.29	0.40	2.06
**19**	0.86	0.60	2.43	**42**	0.54	0.50	4.75
**20**	0.79	0.55	6.27	**43**	0.27	0.40	6.15
**21**	0.21	0.35	4.20	**44**	0.05	0.25	6.63
**22**	0.28	0.40	4.69	**45**	0.04	0.20	8.16
**23**	0.14	0.30	5.45				

A 1mm slice thickness was used for all CT scans in this study. The CAVMAT and VMAT plans were recalculated at different DLG values (0.4, 0.8, 1.2 mm), using the same MU as their 0.4 mm DLG plan. The total MU for the CAVMAT and VMAT plans is displayed below in table 2

Table 2: Total MU for the CAVMAT and VMAT plans.

Total MU		
**Plan #**	VMAT	CAVMAT
**1**	5267	7918
**2**	6072	9054
**3**	6169	8085
**4**	6930	8092
**5**	7810	11402
**6**	7895	5928
**7**	5527	8799
**8**	5478	5859
**9**	7599	7390
**10**	5559	7233

CAVMAT was previously introduced as an improved VMAT technique which prioritizes intuitive collimation and the elimination of MLC openings and dose bridging between targets[1,2].

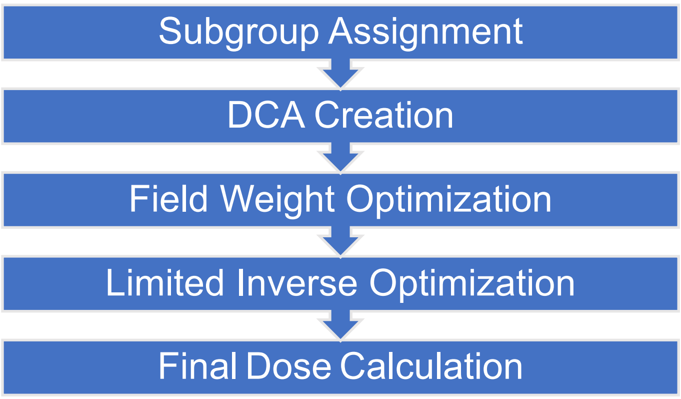

Figure 1: CAVMAT planning process(left); CAVMAT target subgrouping (right),

The CAVMAT technique consists of the following five stages: subgroup assignment, DCA creation, field weight optimization, and limited inverse optimization, after which dose is calculated. The subgroup assignment stage involves separating all targets of a plan into subgroups based on particular target criteria, for each gantry and couch angle. For the first subgroup of an arc, the first target may be selected at random. Subsequent targets follow the subgrouping criteria and may be added to the subgroup if a collimator angle can be found to completely collimate between targets, target superposition does not occur, and that the average effective depths of the targets are not substantially different.

Target superposition is considered and avoided in the subgrouping process as it may lead to dose bridging between targets[3]. Lastly, when all other criteria have been fulfilled, subgroup uniqueness is also prioritized as subgroup variability assists in the subsequent field weight optimization, The subgrouping criteria work in conjunction to minimize dose bridging between targets and to prevent large dose disparities that may be difficult to mitigate in the field weight optimization.

The dose per MU to the center of each target is calculated for all sub-arcs, and the field weighting of all sub-arcs is optimized to achieve uniform dose and MU across all sub-arcs. Iterative field weight optimization is performed in MATLAB using a Nelder-Mead minimization algorithm[4]. Limited VMAT inverse optimization is then performed using Eclipse version 15.6 (Varian Medical Systems, Palo Alto, CA)[5] using the Photon Optimization (PO) algorithm 15603. The optimization is restricted to multi-resolution (MR) level 4 to constrain the optimization to small, fine-tuned MLC adjustments[(5,6)].

The relative complexity of CAVMAT can be assessed and compared against VMAT using modulation complexity scores (MCS). Eclipse API scripting was used to implement a MCS script for the 10 VMAT and CAVMAT plans featured in this study[7]. The MCS script was used to determine the overall complexity and equivalent field sizes of the plans, with a constant DLG setting of 0.4 mm. The MCS implementation evaluates the leaf sequence variability (LSV) and aperture area variability (AAV) to assess variation in MLC position, field shape irregularity, and field area. MCS values range from 0 to 1, with an open, non-modulated field receiving a score of 1.0 and a highly modulated field receiving scores approaching 0[8].

The VMAT plans demonstrated an average MCS score of 0.04±0.02 for an average equivalent field size of 1.51±0.40 cm^2. In comparison, the CAVMAT plans were less modulated, with an average MCS score of 0.05±0.03 for a smaller average field size of 1.11±0.40 cm^2.

Figure 2 below illustrates the dosimetric impact of varying the DLG from 0.4 mm to 0.8 mm and 1.2 mm, for a sample target planned with VMAT and CAVMAT.

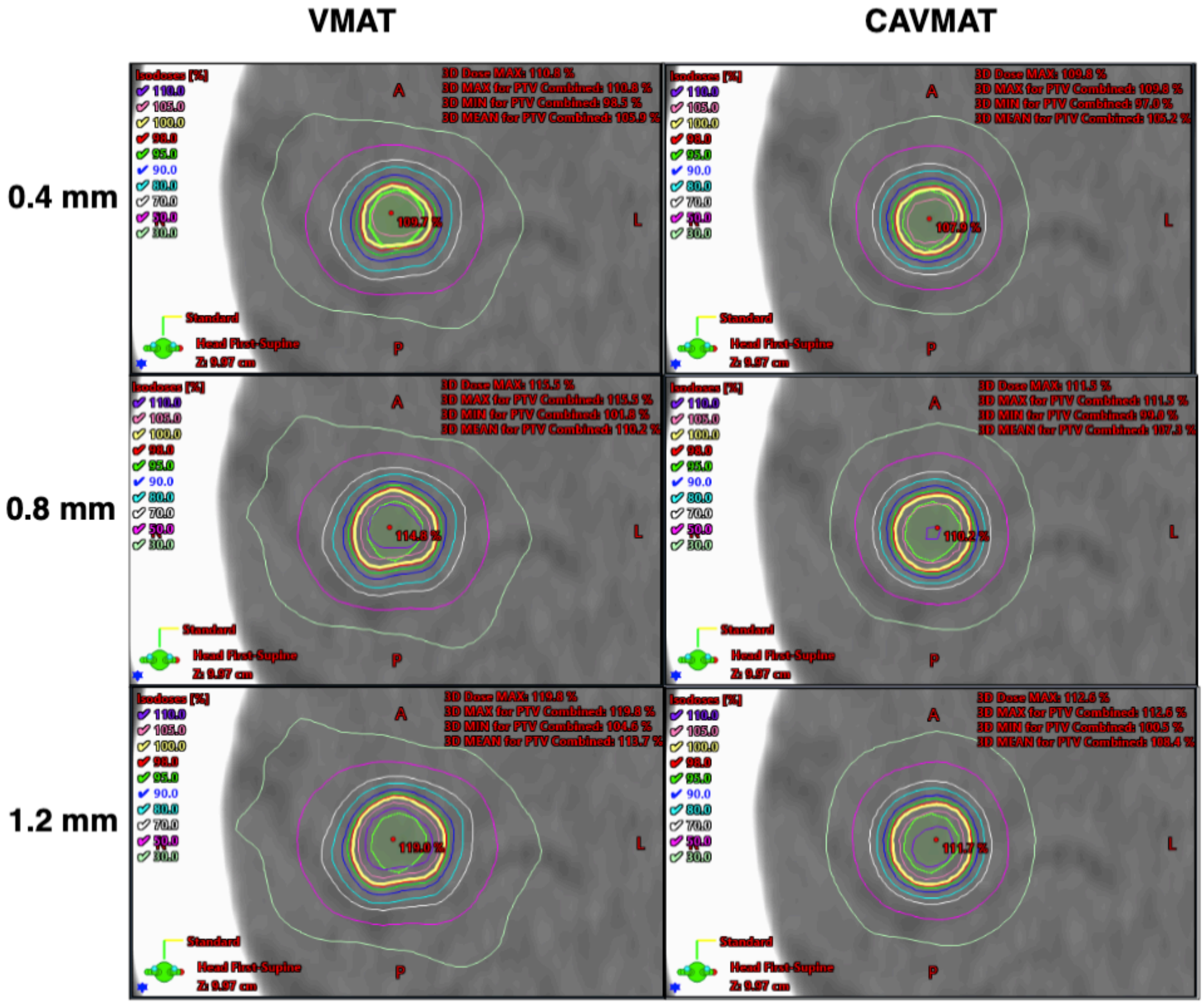

Figure 2: Change in isodose lines of VMAT and CAVMAT for DLG settings of 0.4 mm, 0.8 mm, and 1.2 mm

Figure 3 below illustrates the effect of DLG change on the DVH for the same target.

DLG values are from left to right, 0.4 mm, 0.8 mm, and 1.2 mm.

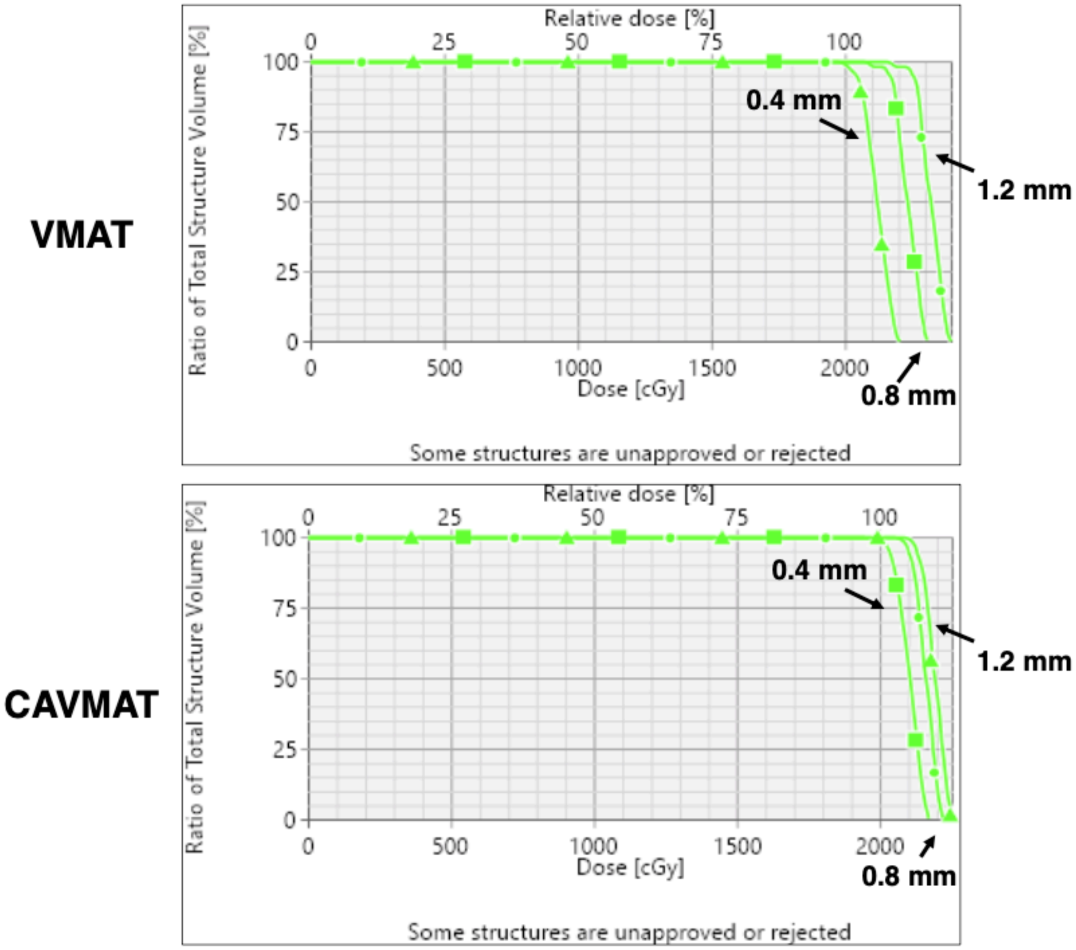

Figure 3: Change in DVH with DLG value for CAVMAT and VMAT

---

[1] E. Cullom, J. Adamson, O. Laryea, J. Kirkpatrick, F.F. Yin, and W. Giles, Improved Radiosurgery Treatment Planning Using Conformal Arc Informed Volumetric

Modulated Arc Therapy [abstract]. In: American Association of Physicists in Medicine 61st annual meeting; 2019 July 14-18; San Antonio, Texas: 2019.

[2] Accepted, in press: Giles WM & Cullom ET, Laryea OA, et al. Radiosurgery treatment planning using conformal arc informed volumetric modulated arc therapy. 2020;():1-10. doi:10.1016/j.meddos.2020.06.00

[3] Kang, J., Ford, E. C., Smith, K., Wong, J., & McNutt, T. R. (2010). A method for optimizing LINAC treatment geometry for volumetric modulated arc therapy of multiple brain metastases. Medical Physics, 37(8), 4146–4154. https://doi.org/10.1118/1.3455286

[4] J. A. Nelder, R. Mead, A Simplex Method for Function Minimization, The Computer Journal, Volume 7, Issue 4, January 1965, Pages 308–313, https://doi.org/10.1093/comjnl/7.4.308

[5] Varian Medical Systems. (2017). Eclipse Photon and Electron Algorithm Reference Guide (April), 263-348

[6] Liu, H., Sintay, B., Pearman, K., Shang, Q., Hayes, L., Maurer, J., … Wiant, D. (2018). Comparison of the progressive resolution optimizer and photon optimizer in VMAT optimization for stereotactic treatments. *Journal of Applied Clinical Medical Physics*, *19*(4), 155–162. https://doi.org/10.1002/acm2.12355

[7] Ohira, Shingo, et al. "HyperArc VMAT planning for single and multiple brain metastases stereotactic radiosurgery: a new treatment planning approach." Radiation Oncology 13.1 (2018): 13.

[8] McNiven, A.L., Sharpe, M.B. and Purdie, T.G. (2010), A new metric for assessing IMRT modulation complexity and plan deliverability. Med. Phys., 37: 505-515. doi:10.1118/1.3276775
